# Isoconversional Analysis of the Catalytic Pyrolysis of ABS, HIPS, PC and Their Blends with PP and PVC

**DOI:** 10.3390/polym16162299

**Published:** 2024-08-14

**Authors:** Maria-Anna Charitopoulou, Evangelia C. Vouvoudi, Dimitris S. Achilias

**Affiliations:** Laboratory of Polymers and Colours Chemistry and Technology, Department of Chemistry, Aristotle University of Thessaloniki, GR-54124 Thessaloniki, Macedonia, Greece

**Keywords:** WEEE, thermal degradation, activation energy, catalytic cracking, plastics recycling

## Abstract

Thermochemical recycling of plastics in the presence of catalysts is often employed to facilitate the degradation of polymers. The choice of the catalyst is polymer-oriented, while its selection becomes more difficult in the case of polymeric blends. The present investigation studies the catalytic pyrolysis of polymers abundant in waste electric and electronic equipment (WEEE), including poly(acrylonitrile-butadiene-styrene) (ABS), high-impact polystyrene (HIPS) and poly(bisphenol-A carbonate) (PC), along with their blends with polypropylene (PP) and poly(vinyl chloride) (PVC). The aim is to study the kinetic mechanism and estimate the catalysts’ effect on the activation energy of the degradation. The chosen catalysts were Fe_2_O_3_ for ABS, Al-MCM-41 for HIPS, Al_2_O_3_ for PC, CaO for Blend A (comprising ABS, HIPS, PC and PP) and silicalite for Blend B (comprising ABS, HIPS, PC, PP and PVC). Thermogravimetric experiments were performed in a N_2_ atmosphere at several heating rates. Information on the degradation mechanism (degradation steps, initial and final degradation temperature, etc.) was attained. It was found that for pure (co)polymers, the catalytic degradation occurred in one-step, whereas in the case of the blends, two steps were required. For the estimation of the activation energy of those degradations, isoconversional kinetic models (integral and differential) were employed. In all cases, the catalysts used were efficient in reducing the estimated *E*_α_, compared to the values of *E*_α_ obtained from conventional pyrolysis.

## 1. Introduction

This is the third decade where the economic and technological advances, in combination with the upgraded living conditions, result in a considerable increase in the consumption of electric and electronic equipment (EEE) [1] and, therefore, in huge amounts of post-consumer electronic devices. Their short lifespan and non-biodegradability are the main reasons for the accumulation of such wastes in the environment, which, however, are not usually toxic but rather undesirable due to their prolonged degradation [2]. Thus, EU legislation has become stricter over the years, forcing research to be focused on finding less-energy-consuming approaches for their management while contributing to a circular economy [3]. Until recently, the disposal of waste electric and electronic equipment (WEEE) has been taking place via land-filling, primary recycling or mechanical recycling, but today, energy recovery or chemical recycling is promoted [2,4]. In the case of land-filling, large expanses of ground are occupied and polluted with wastes, resulting in possible infection of the groundwater because of contaminants’ leaching [5].

Nevertheless, the recycling of WEEE is challenging since they consist of plenty of materials, such as metals, glass and plastics, some of which could be reused [6]. Many types of (co)polymers can be identified in the plastic content of WEEE, including poly(acrylonitrile-butadiene-styrene) (ABS), high-impact polystyrene (HIPS), poly(bisphenol-A carbonate) (PC), polypropylene (PP), poly(styrene-acrylonitrile) (SAN), etc., as well as their blends [7]. Moreover, one main obstacle in the recycling of polymers found in WEEE is the fact that they often include heavy metals (e.g., Pb, Hg, Cd and Cr^6+^) along with harmful additives, namely plasticisers, colourants, antistatic agents, flame retardants (such as brominated flame retardants (BFRs)), etc. [8,9,10].

In the case of primary recycling, wastes must be uncontaminated and of a single type in order to be re-introduced into the heating cycle [3,11]. Likewise, in mechanical recycling, wastes have to be homogeneous unless sorting and separating processes proceed. Furthermore, mechanical recycling leads to a deterioration of the product’s properties in every recycling cycle [3], and each polymer can withstand only a limited number of reprocessing cycles [12]. Energy recovery is often unacceptable, since in the event that incomplete incineration of WEEE happens, many toxic substances, such as dioxins, polychlorinated biphenyls and furans, can be formed and released in the atmosphere [2]. This is why thermochemical recycling, such as pyrolysis, is often selected by researchers as an environmentally friendly method since secondary valuable materials can be produced while the liquid fraction may be used as fuel for energy production [2,3]. Chemical or tertiary recycling is a technique in which plastic waste is converted into products of lower molecular weight through chemical reactions by using solvents and reagents (solvolysis and thermolysis) [13,14]. The pyrolysis of waste polymers converts them into liquid oil, char and gases via thermal decomposition after being treated at high temperatures (300–900 °C) and in an inert atmosphere [13,15]. Pyrolysis can take place in the absence or presence of catalysts. The quality of the derived pyrolysis products and their distribution depends on various parameters, such as the type of the reactor, the form of the waste and, especially, the operating conditions (temperature, pressure, residence time, heating rate, type of catalysts) [16]. Among these parameters, temperature seems to play the most important role regarding the products’ distribution, since a high amount of liquid oil (up to 80 wt%) may be yielded at moderate temperatures around 500 °C and with fast (flash) heating rates [17]. Slow heating rates allow time for further degradation into gas products at the same or a bit lower pyrolysis temperatures.

Catalysts, known to increase the conversion rate at lower degradation temperatures and shorter reaction times compared to conventional decomposition, reduce energy requirements and the residual percentage [6]. The internal porous structure of a heterogenous catalyst provides channels for the selective movement and cleavage of larger compounds into smaller ones. The recovery of the catalyst is difficult after direct contact with the polymer since it is hindered due to the sticky nature of the plastic raw material. The main advantages of Lewis acid catalysts include resistance and morphology, such as BET surface area, acidity, pore size and volume, Si/Al ratio, thermal stability and dimensions. Starting with silicalite, it has been reported that the morphology of zeolite can be controlled using different solvents, a variable source of silica and a moulding agent. The incorporation of Al and transition metals into the silica structure has been applied to increase the acidity, ion-exchange capacity and overall catalytic activity [18]. On the other hand, studies of Al-MCM-41 (a mesoporous material with a hierarchical structure from the family of aluminosilicates) with different Si/Al ratios are reported, where the content of silanol groups is dependent on the synthesis methods or its clarification [19]. Although Al-MCM-41 is a useful catalyst in organic reactions, the aluminosilicate framework is not similar to that in zeolites but rather amorphous, with a larger pore diameter than that of zeolites. The degree of condensation of the aluminosilicate in the context of Al-MCM-41 reduces both the acidity and the hydrothermal stability of Al-MCM-41 [20]. Many researchers have studied CaO as a strong candidate for a heterogeneous catalyst due to its commercial availability, economic advantage and relatively higher activity [21]. Fe_2_O_3_ is a ferromagnetic substance, dark red in colour and easily attacked by acids [22]. *γ*-Al_2_O_3_ illustrates the desired properties, such as sufficient surface, pore volume and pore size distribution, as well as acid/base characteristics mainly due to its local microstructure [23].

Due to pyrolysis’s advantages, many researchers have applied chemical recycling methods even on real waste originating from WEEE, including printed circuit boards (PCBs), tablets and mobile phones, wires, etc., in an attempt to find the appropriate experimental conditions so as to obtain products that could lead to valuable chemicals or bio-fuels [24,25,26]. In order to design and set the appropriate parameters for a pyrolysis process, it is vitally important to study the mechanisms of the reactions that occur and, so, to understand more deeply the kinetics of the thermal or catalytic decomposition of the material examined. As a result, research has focused on the thermal (mainly [27,28,29]) and catalytic degradation kinetics of various polymers found in WEEE [6,30]. Specifically, Durmus et al. [30] examined the catalytic degradation of PP, free of any kind of additives, using zeolite catalysts (like BEA, ZSM-5 and MOR types) with different properties, such as surface areas, pore structures, acidities and Si/Al molar ratios. In order to estimate the degradation rate of PP over zeolites, they applied thermogravimetric analysis (TGA) employing four different heating rates to calculate the activation energies according to the Kissinger equation [30]. Siddiqui et al. [6] investigated the pyrolysis kinetics of PC by conducting TGA experiments while applying different heating rates and estimated the activation energy using the Wang or Friedman methods [6].

Surely, polymers’ degradation in an inert or oxidative atmosphere includes changes in the chemical structure of the chains, molecule abstraction, group elimination or random scission. TGA records the mass loss profile over rising temperatures. As regards the derivative curves, they offer deeper information on the polymers’ degradation steps since they estimate the number and shape of the peaks formed. In TG curves, slope changes are indicative of the degradation parameters. TGA is preferred since it examines the overall thermal degradation kinetics of a polymer by giving out information about the activation energy, the exponential factor and the total reaction order [31,32].

In the present investigation, experiments of catalytic pyrolysis of ABS, HIPS, PC and their blends with PP and PVC were carried out, since they are the most abundant plastics found in WEEE. Recently, the thermal degradation kinetics of ABS, HIPS, PC and their blends with PP and PVC have been published, along with the analysis of pyrolyzates eluted from catalytic experiments [33,34]. As a step further, the kinetics of catalytic pyrolyses of the mentioned polymers and blends are investigated here in order to find ways for their effective thermal degradation. Therefore, the aim of this work was to study the kinetic profile and mainly estimate the effect of the catalysts on the activation energies of the degradation. The catalysts chosen for this purpose were Fe_2_O_3_ for ABS, Al-MCM-41 for HIPS, *γ*-Al_2_O_3_ for PC, CaO for the first blend (**Blend A** (ABS, HIPS, PC and PP)) and silicalite for the second one (**Blend B** (ABS, HIPS, PC, PP and PVC)). The choice of each catalyst used for each polymer/blend was made after trying, in our previous studies, many catalysts for every single polymer/blend in order to find the best one for each case examined [34]. Lewis acid sites define catalyst acidity; the higher the acidity, the greater the enhancement in the cracking process and the production of gasoline hydrocarbons in the liquid oil [34]. In order to investigate thermal degradation kinetics, thermogravimetric experiments were performed at four different heating rates to gather experimental data and perform isoconversional analyses. Our results are proven useful since the catalysts suggested here may be applied in the catalytic pyrolysis of real WEEE in order to reduce energy consumption during their thermal degradation.

## 2. Materials and Methods

### 2.1. Materials

The following polymers used in the experiments were commercially acquired: ABS [(C_15_H_17_N)_n_, FW = 211.3, batch# 01519EB, melt index: 6 g/10 min]; HIPS [(C_8_H_8_)_x_∙(C_4_H_6_)_z_, CAS 9003-55-8, lot# 02122CEV, melt index: 6 g/10 min, butadiene content 4%]; PC [(C_15_H_16_O_2_)_n_, CAS 25037-45-0, lot# 07624KHV, melt index: 7 g/10 min]; *iso*-PP ([CH_2_CH(CH_3_)]_n_, CAS 9003-07-0, batch# 04227KC); and PVC [(C_2_H_3_Cl)_n_, M_n_ = 47,000, M_w_ = 80,000] (all supplied by Sigma-Aldrich^®^ (MO, USA)). All polymer pellets were grounded using an Arthur H. Thomas Co. mill (PA, USA) to reduce particle size (~0.2 mm) and enable surface augmentation for the pyrolysis experiments. As mentioned in the Introduction, two polymeric blends, Blend A and Blend B, were prepared with the solution-casting technique as described in Ref. [33]. **Blend A** consisted of 35% ABS, 25% HIPS, 15% PC and 25% PP, and **Blend B** consisted of 37% ABS, 20% HIPS, 7% PC, 30% PP and 6% PVC. Flakes were boiled in reflux of PhCOCl for 6 h and then cast in an open vessel for solvent removal in ambient conditions. Their percentages were based on the literature data [35,36].

The catalysts that were studied included the following solid (Lewis acid) materials: a transition metal oxide, Fe_2_O_3_, for pyrolysis of ABS; a mesoporous alumino-silicate, Al-MCM-41, for pyrolysis of HIPS; an alumina, *γ*-Al_2_O_3_, for pyrolysis of PC; and a representative zeolite, silicalite, for pyrolysis of Blend B. A Lewis base catalyst, CaO, was also used for pyrolysis of Blend A. All catalysts were used as received in powder form after moisture drying. Information on their characteristics can be found in Ref. [37].

### 2.2. Thermogravimetric Analysis (TGA)

The pyrolysis experiments were conducted on a Pyris 1 TGA thermal analyser (Perkin Elmer, Norwalk, CT, USA) equipped with Pyris Software v.3.81 (rev. in 2000) for Windows. The samples weighted about 6 mg (balance accuracy 0.0001 mg) and were placed in a Pt crucible, then mixed thoroughly with a little spatula. The specimens were first held at 40 °C for 1 min (for temperature homogeneity through the mass) and then heated from 40 to 700 °C (thermostat accuracy ± 2 °C) at four different heating rates (*β* = 2.5, 5, 10 and 20 °C·min^−1^). The polymer/catalyst mass ratio was 2:1. The sample mass loss was plotted over temperature and recorded continuously. The residue was of grey colour, black for polymer traces and unchangeable for catalyst powders.

## 3. Theoretical Background on the Kinetic Analysis

The kinetic analysis of polymer degradation can be carried out by recording the reduction in sample mass with either increasing temperature (non-isothermal experiments) or increasing time (experiments under constant temperature). The extent of the degradation (*α*) can be calculated from Equation (1):

(1) $$\alpha =\frac{m_{0}-m_{t}}{m_{0}-m_{f}}$$

where *m_0_*, *m_t_* and *m_f_* refer to the polymer mass at the beginning of the degradation, at any time (*t*) and at the end of the degradation, respectively [6,31]. 

The kinetics of polymer degradation can be described by the following single-step kinetic equation (Equation (2)) that expresses the variation in the degree of degradation (*α*) with time (*t*) and temperature (*T*) in K [38]:

(2) $$\frac{d\alpha }{dt}=k\left(T\right)\cdot f\left(\alpha \right)$$

In Equation (2), *k*(*T*) is the temperature-dependent rate constant, and *f*(α) is the function that expresses the reaction model. The temperature-dependent rate constant is typically expressed through the Arrhenius equation (Equation (3)):

(3) $$k\left(T\right)=A\;\cdot \;e^{\left(-\frac{E_{\alpha }}{RT}\right)}$$

As a result, Equation (2) becomes

(4) $$\frac{d\alpha }{dt}=A\;\cdot \;e^{\left(-\frac{E_{\alpha }}{RT}\right)}\;\cdot \;f\left(\alpha \right)$$

In Equation (4), *A* and *E_α_* are the kinetic parameters that represent the pre-exponential factor and the activation energy, respectively. *R* is the universal gas constant. It should be mentioned that, at a constant extent of conversion, the reaction rate is a function only of the temperature, and so Equation (4) can be converted into Equation (5) [39]:

(5) $${\left[\frac{dln\left(d\alpha /dt\right)}{d\left(1/T\right)}\right]}_{\alpha }=-\frac{E_{\alpha }}{R}$$

Isoconversional methods employ multiple temperature programs (e.g., different heating rates or temperatures) so as to obtain data on varying rates at a constant extent of conversion. In addition, these methods allow complex (i.e., multi-step) processes to be detected via a variation in *E_α_* with *α* [31,39]. Isoconversional methods can be divided into two categories: differential and integral. The Friedman method is the most common differential isoconversional method [38], based on Equation (6):

(6) $$\ln{\left(\frac{d\alpha }{dt}\right)}_{\alpha ,i}=\ln\left[f\left(\alpha \right)A_{a}\right]-\frac{E_{\alpha }}{RT_{\alpha ,i}}$$

At any given *α*, the value of *E_a_* can be determined by the slope of a plot of ln(dα/dt) vs. 1/*T*. Since this method does not use any kind of approximations, it can potentially be more accurate than integral methods [38]. Nevertheless, the Friedman method requires numerical differentiation of the experimental *α* vs. *T* curves, which is usually carried out by the software of the instrument. Unfortunately, more often than not, the latter results in receiving noisy rate data and, consequently, unstable activation energy values [6,39]. 

However, there is no such thing as numerical differentiation in integral methods, so by applying them, the problem may be overcome. For non-isothermal conditions, when the temperature is raised at a constant heating rate (*β = dT/dt*), the integration of Equation (4) results in *g*(*a*), a function that indicates the integral form of the reaction model (Equation (7)):

(7) $$g\left(\alpha \right)=\int_{0}^{\alpha }\frac{d\alpha }{f\left(\alpha \right)}=A\int_{0}^{t}e^{-E_{\alpha }/\left(RT\right)}dt=\frac{A}{\beta }\int_{{Τ}_{0}}^{{Τ}_{a}}e^{-E_{\alpha }/\left(RT\right)}dT=\frac{A}{\beta }\;I\left(E,T\right)$$

Equation (7) does not have any analytical solution, so it can be solved by using either approximations or numerical integration. 

One of the simplest approximations, made by Doyle, results in Equation (8), which is used in the isoconversional methods of Flynn, Wall and Ozawa [39,40,41,42]:

(8) $$\ln(\beta_{i})=Const.-\frac{1.052E_{\alpha }}{RT_{\alpha ,i}}$$

A more precise approximation by Coats and Redfern leads to Equation (9), known as the Kissinger–Akahira–Sunose (KAS) equation [39,43]:

(9) $$ln\left(\frac{\beta_{i}}{T_{\alpha ,i}^{2}}\right)=constant-\frac{E_{\alpha }}{RT_{\alpha ,i}}$$

The activation energy (*E_α_*) can be calculated from the slope of the curves received after plotting *ln*(*β/T*^2^) vs. 1/*T*. The KAS equation offers more accuracy as regards the *E_α_* values than those calculated from the Ozawa–Flynn–Wall method [38]. In the present work, the isoconversional methods chosen for the calculation of *E_α_* were both the **Friedman** and the **KAS** methods.

## 4. Results and Discussion

**Figure 1:**
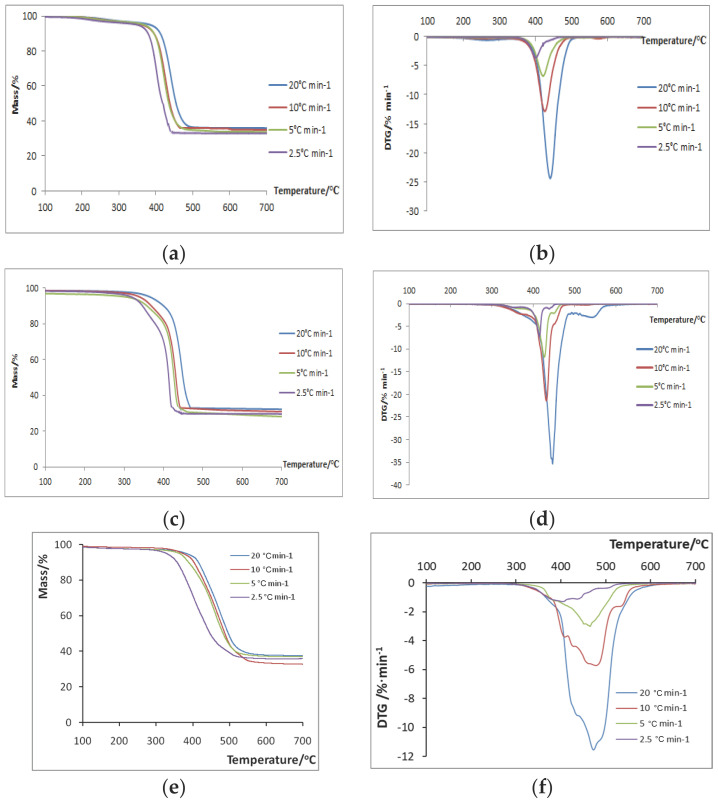
Charts of mass loss vs. temperature for (**a**) ABS/Fe_2_O_3_ samples, (**c**) HIPS/Al-MCM-41 samples and (**e**) PC/Al_2_O_3_ samples. Charts of differential TG curves vs. temperature for (**b**) ABS/Fe_2_O_3_ samples, (**d**) HIPS/Al-MCM-41 samples and (**f**) PC/Al_2_O_3_ samples.

The TG curves, as well as their derivative DTG curves, for the four different heating rates for ABS/Fe_2_O_3_, HIPS/Al-MCM-41 and PC/Al_2_O_3_ are presented in Figure 1a–f, respectively. From Figure 1a,c,e, it is noticeable that as the heating rate *β* is decreased from 20 to 2.5 °C·min^−1^, the mass loss curves and the degradation temperatures shift to lower values, something that is expected in all cases [31]. According to the DTG curves shown in Figure 1b,d,f, maximum degradation occurs at temperatures above 400 °C and follows a one-step mechanism. In Figure 1d, there are two peaks in the DTG curves. The first sharp peak displays the maximum degradation loss and is considered the major degradation step, while the second peak is much weaker and is considered a secondary phenomenon. 

Similarly, in Figure 1e, the decrease in heating rate leads to lower values of mass residuals and degradation peak temperatures, while the maximum mass loss occurs at temperatures greater than 400 °C again. According to their DTG curves (Figure 1f), there are multiple peaks at all rates examined. At a rate of 20 °C·min^−1^, the first peak (~430 °C) and the third peak (~490 °C) are considered shoulders since they are much weaker than the main peak at ~470 °C, which seems to be the major degradation step. This is in accordance with the literature reports on the DTG curves of neat PC, where, again, degradation occurs in one stage [44]. Likewise, for the other rates, the main peaks were recorded at ~480 °C for 10 °C·min^−1^, ~465 °C for 5 °C·min^−1^ and ~400 °C for 2.5 °C·min^−1^.

**Figure 2:**
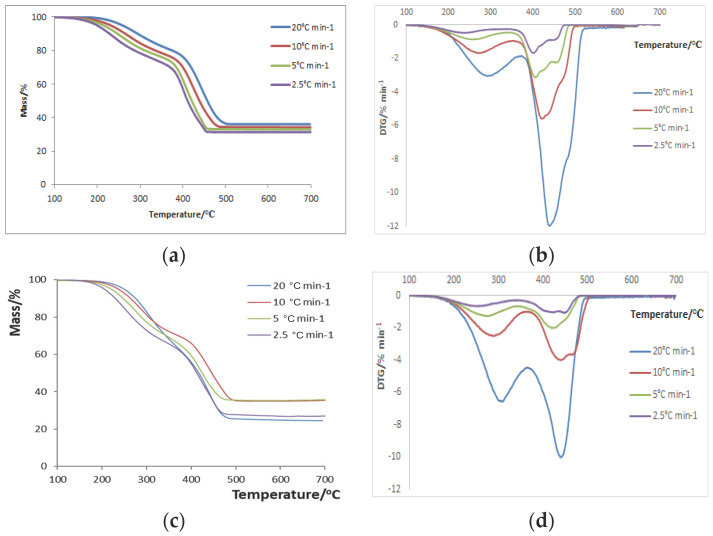
Charts of mass loss vs. temperature for (**a**) Blend A/CaO samples and (**c**) Blend B/silicalite samples. Charts of differential TG curves vs. temperature for (**b**) Blend A/CaO samples and (**d**) Blend B/silicalite samples, respectively [33,35,36].

In Figure 2a–d, the TG and DTG curves for the Blend A/CaO samples and the Blend B/silicalite samples are shown, respectively. It may be noticed that in the case of the blends, the decrease in heating rate also results in lower values of mass residuals and degradation peak temperatures. Nevertheless, in the case of the blends, the catalytic degradation occurs in two steps, in contrast to the catalytic degradation of neat polymers [33]. This may be attributed to the blends’ composition, which can affect the degradation behaviour since various interactions can take place among the blends’ components and among the degradation products [45].

All the reactions taking place can either result in an acceleration of the degradation process or in a stabilising effect, compared to the reactions observed in non-catalytic experiments [45]. The first degradation step is smaller than the second one and appears at temperatures >300 °C, with mass losses of ~10–15%. The second (main) degradation step occurs at temperatures of ~400–440 °C, with greater mass losses of ~40–60%, depending on the heating rate applied. Moreover, as the heating rate increases, the peaks become more intense. In Table 1, the temperature and mass loss values are listed in detail for the initiation, maximum and end of the degradation for each sample and each rate applied. T_in_ and T_fin_ were determined by drawing tangents at the TG curves, which were examined visually.

As regards the maximum degradation temperature, it is noticed that the T_peak_ of PC/Al_2_O_3_ at almost all rates is greater than that of the other polymer/catalyst combinations, something that is quite expected due to the high thermal stability of PC itself [33,37]. It should also be highlighted that T_in_, for both blend/catalyst combinations and for each heating rate examined, is lower than that of the copolymer/catalyst combinations. This happens due to a synergistic effect of the blends’ components and their degradation products (e.g., the radicals that are formed, etc.) that could interact with the polymeric structure, resulting in an acceleration of the degradation rate [45]. In addition, it should be mentioned that, in almost all cases, the presence of catalysts shifts T_peak_ towards lower values than those identified for neat polymers or blends; thus, the catalyst plays a key role in energy diminution. For instance, in the case of neat PC, the T_peak_ values were 543, 528, 511 and 492 °C for 20, 10, 5 and 2.5 °C·min^−1^, respectively [33], whereas now, according to Table 1, they are 473, 481, 465 and 402 °C, respectively. As a result, it can be concluded that catalysts were efficient in accelerating the degradation by resulting in lower degradation temperatures compared to non-catalytic decompositions for such a thermally durable polyester. 

Regarding residual mass, it should be pointed out that, according to the experimental plan, 67% of the total mass corresponds to the polymer, whereas 33% corresponds to the catalyst. Thus, residual masses lower than expected are attributed to possible condensation reactions taking place in inorganic oxides, since the -OH groups that exist in the crystals (even as complexes or ligands) may be affected at high temperatures [31]. In a polymer such as PC, residues with stiff aromatic chains are produced, resulting in a char content of 25–27% without a catalyst [33], but as Table 1 indicates, the specific combination for PC promotes organic decomposition into secondary products and less char remains. 

**Figure 3:**
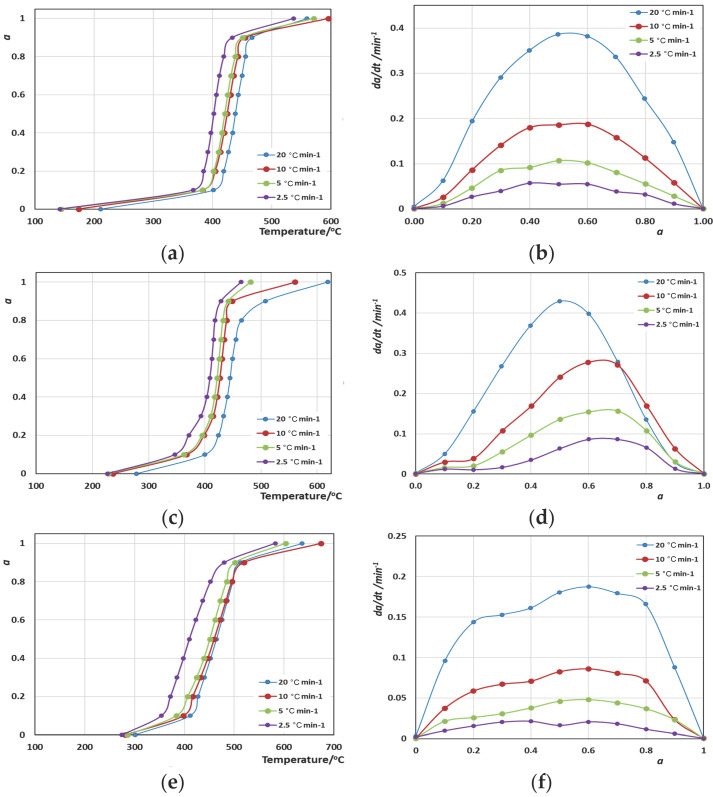
Variation in (**a**) *α* vs. *T* and (**b**) d*α*/d*t* vs. *α* curves for ABS/Fe_2_O_3_; (**c**) *α* vs. *T* and (**d**) d*α*/d*t* vs. *α* curves for HIPS/Al-MCM-41; and (**e**) *α* vs. *T* and (**f**) d*α*/d*t* vs. *α* curves for PC/Al_2_O_3_, respectively.

**Figure 4:**
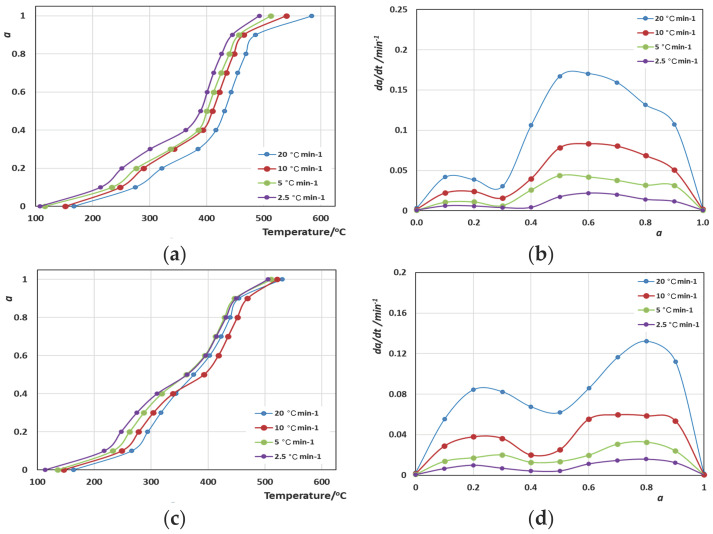
Variation in *α* vs. *T* (**a**) and d*α*/d*t* vs. *α* curves (**b**) for Blend A/CaO; and *α* vs. *T* (**c**) and d*α*/d*t* vs. *α* curves (**d**) for the Blend B/silicalite systems.

Moving on to the calculation of the activation energies, the methods that were used included the integral KAS method and the differential Friedman method. The determination of *α* took place considering that *α* = 0 when *T* = *T*_in_ and *α* = 1 when *T* = *T*_fin_. The use of Equation (9) for the KAS method and Equation (6) for the Friedman method led to the estimation of the evolution of the activation energy (*E_α_*) values with the extent of the conversion of the reaction (*α* refers to the extent of the degradation here). It should be mentioned that the straight lines *ln*(*β*/*T*^2^)-1/*Τ* and *ln*(d*a*/d*t*)-1/*T* obtained were very good in almost all cases, presenting an R^2^ > 0.9. For the calculation of *E_α_*, first, the calculation of *α* took place. So, in Figure 3a–f and Figure 4a–d, the curves of the variation in *α* vs. *T* and d*a*/d*t* vs. *a* for all polymers and blended materials are shown.

The curves shown in Figure 3b,d,f show the same pattern that verifies the observations regarding the single-step catalytic decomposition, with rates (d*a*/d*t*) being twice or three times higher when *β* = 20 °C·min^−1^ compared to 10 °C·min^−1^. Similar charts in Figure 4b,d present a double-step decomposition: for Blend A, the first step extends to *a* = 0.3, while for Blend B, the first step extends to *a* = 0.5, which makes Blend B a less durable material. We attribute this weakness not to the composition percentage differences of stiff ingredients such as PC, but mostly to the presence of PVC, which heavily promotes radical decomposition reactions.

**Figure 5:**
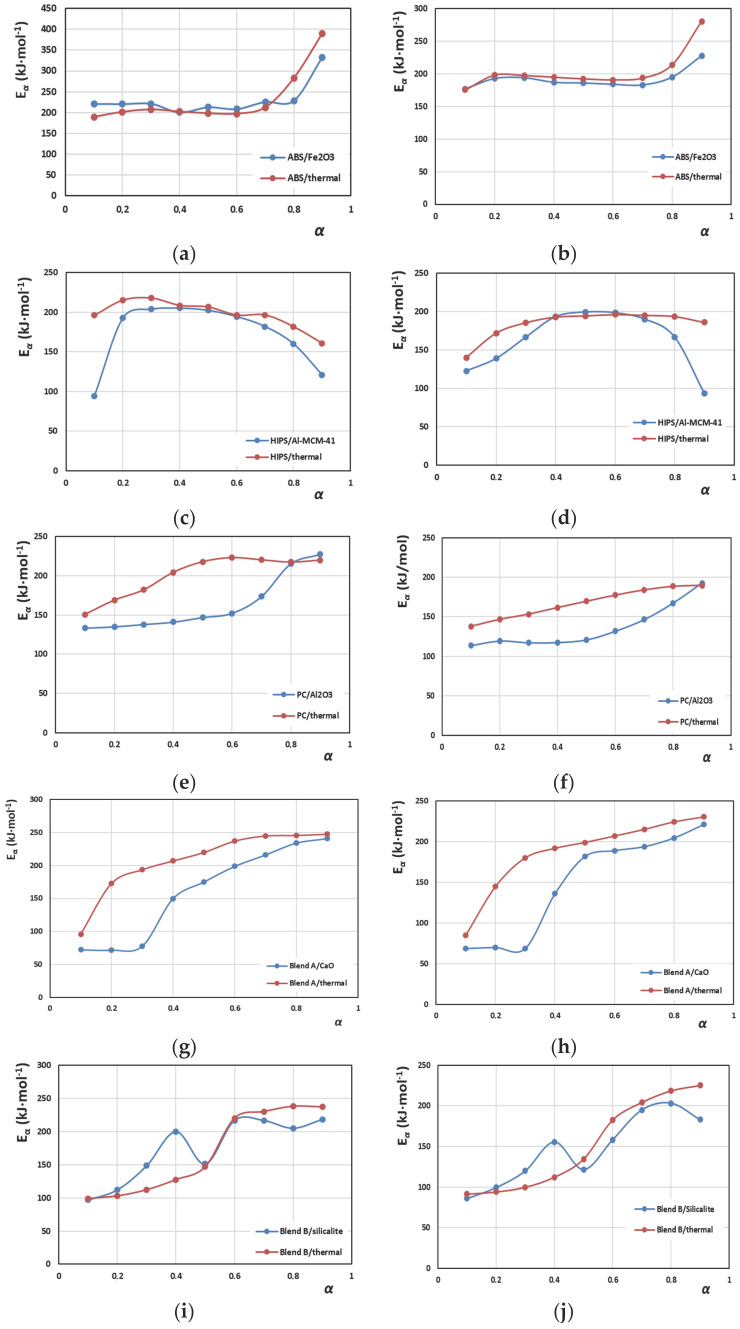
Comparisons of the effect of the catalyst on the variation in the activation energy (*E_α_*) with the extent of degradation (*α*) for (**a**) ABS/Fe_2_O_3_ using the Friedman method, (**b**) ABS/Fe_2_O_3_ using the KAS method, (**c**) HIPS/Al-MCM-41 using the Friedman method, (**d**) HIPS/Al-MCM-41 using the KAS method, (**e**) PC/Al_2_O_3_ using the Friedman method, (**f**) PC/Al_2_O_3_ using the KAS method, (**g**) Blend A/CaO using the Friedman method, (**h**) Blend A/CaO using the KAS method and, finally, (**i**) Blend B/silicalite using the Friedman method and (**j**) Blend B/silicalite using the KAS method.

Next, in Figure 5, the activation energy values estimated using two isoconversional methods, namely KAS and Friedman, are illustrated, together with the corresponding values from the literature [33], without using a catalyst. Comparing the results from the two methods, it may be postulated that the trend in the dependence of the activation energy on the extent of degradation *a* was generally similar. The main difference is that the values obtained from the Friedman calculations (differential method) are shifted to slightly higher values compared to those from the KAS calculations (integral method). This difference is attributed to the specific characteristics of the methods employed and has long and deeply been discussed in the literature [29]. Furthermore, in most cases, *E_α_* is low when 0.1 < *α* < 0.3, which is attributed to the fact that degradation is less energy-demanding at the beginning of pyrolysis due to the elimination of some weak groups in the macromolecular structures. Later on, after the formation of macro-radicals, the degradation continues through various radical pathways (*β*-scission, H-abstraction, unzipping, disproportionation), resulting in variations in *E_α_* values [29,46]. Specific comments follow for each catalytic system.

For ABS/Fe_2_O_3_ and for the range of 0.2 < *α* < 0.8, *E_α_* values varied between 183 and 195 kJ·mol^−1^ in the case of the KAS method, whereas they varied between 200 and 226 kJ·mol^−1^ in the case of the Friedman method. So, *E_α_* is nearly constant in the range 0.2 < *α* < 0.8 for both methods. This observation, according to the literature, may be indicative of the fact that, from a kinetic point of view, the degradation is a single-step process [47,48], which is in accordance with the results obtained from the DTG curves (Figure 1a,b and Figure 3a,b). As regards the effect of the catalyst on *E_a_*, it is worth mentioning that until *α* = 0.7, the values are similar, whereas at higher conversions, and mainly at *α* = 0.9, the presence of Fe_2_O_3_ results in lower activation energies for both methods applied. Specifically, for pure ABS, *E_a_*_,max_ was found to be 280 kJ·mol^−1^ in the case of the KAS method and ~390 kJ·mol^−1^ in the case of the Friedman method, according to our previous publication [33], whereas now, in the presence of Fe_2_O_3_, the maximum values were ~228 and ~333 kJ·mol^−1^ for the KAS and Friedman methods, respectively. Similarly, the literature finds that the random scission model is a suitable mechanism to describe the thermal degradation of ABS plastic waste.

For the HIPS/Al-MCM-41 system, the *E_a_* curve’s shape is much different. At first, *E_a_* increases with *α*, reaches a maximum at *α* = 0.5 (~199 kJ·mol^−1^) for the KAS method and at 0.4 (~205 kJ·mol^−1^) for the Friedman method and then decreases, indicating perhaps a more complex multi-reactional mechanism that involves parallel and/or reversible reactions [48]. The literature states that, apart from the radical decomposition mechanism, an ionic degradation mechanism also occurs for HIPS when oxides are incorporated at high temperatures. The shape of the curve is similar to that observed in the case of the non-catalytic pyrolysis of HIPS, where *E_a_* increased, reached a maximum and then decreased [33]. As for the catalyst’s effect, it was found that the initial values of *E_a_* became much lower in the presence of Al-MCM-41 (123 and 94 kJ·mol^−1^ for the KAS and Friedman methods, respectively), compared to those (~140 and <200 kJ·mol^−1^, respectively) obtained in the case of the neat polymer. As mentioned before, the single-step decomposition is evident in HIPS too, a pattern followed in all styrenic materials [49]. One could say that the ABS and HIPS copolymers act similarly since they have a similar macromolecular structure consisting of styrene and butadiene monomers. Yet, it is obvious here, as in the literature [33], that the acrylonitrile monomer changes the behaviour of ABS severely, reducing its thermal tolerance. The -CN groups elaborated onto macrochains are sensitive sites that are affected by radicals and cause the ABS copolymer to degrade in a constant way from the beginning, unlike HIPS, where higher energy levels need to be achieved for the decomposition to proceed (Figure 5c,d).

For PC/Al_2_O_3_, the *E_α_* values varied between 114 and 121 kJ·mol^−1^ for the range of 0.1 < *α* < 0.5 in the case of the KAS method, whereas they varied between 133 and 139 kJ·mol^−1^ for the range of 0.1 < *α* < 0.4 in the case of the Friedman method. Then, at higher extents of degradation, in both cases, *E_a_* increased to higher values up to 213 and 248 kJ·mol^−1^ for KAS and Friedman, respectively. The initial low values of *E_a_* may be attributed to the fact that PC’s thermal degradation begins rather easily due to the ester groups inherent in the polymer chain. As these groups are consumed, the limiting step of degradation is that of random scission [50]. Similar trends have been reported in the literature [51,52], and the higher values of *E_α_* calculated at larger extents of degradation have been attributed to the stable carbonaceous, aromatic skeleton, which protects the polymer from further degradation. It should be mentioned that, in the presence of Al_2_O_3_, the initial values of *E_a_* became much lower than those in the absence of the catalyst. Specifically, non-catalytically, for *α* = 0.1, *E_a_* was 140 and 150 kJ·mol^−1^ (for the KAS and Friedman methods, respectively), but in the presence of the catalyst, the values became 114 and 133 kJ·mol^−1^. 

In the case of the two blends, things are quite different, since *E_a_* shows two maxima. So, *E_α_* does not follow a trend in the whole range of 0.1 < *α* < 0.9, something that is indicative of the fact that the blends’ degradations involve multi-step reactions [53].

For Blend A/CaO, *E_a_* exhibits an initial plateau and is constant in the range of 0.1 < α < 0.3, since it varies between 68.5 and 70 and between 72 and 78 kJ·mol^−1^ for the KAS and Friedman methods, respectively. Then, it increases up to *α* = 0.5, where it exhibits another plateau, and *E_a_* is almost constant in the range of 0.5 < *α* < 0.7, since it varies between 182 and 193 and between 199 and 206 kJ·mol^−1^ for the KAS and Friedman methods, respectively. As regards the effect of the catalyst on *E_α_*, it should be underlined that it results in reduced values of *E_α_* compared to previous analogous calculations [33] of neat Blend A without any catalyst. The catalytic effect of CaO is obvious. Specifically, in the case of the KAS method and in the presence of CaO, for *α* = 0.1, *E_α_* is 68.5 kJ·mol^−1^, remains constant until *α* = 0.3 and finally increases to *E_α_* = 221 kJ·mol^−1^ for *α* = 0.9. Conversely, without the catalyst, *E_α_* starts at 83 kJ·mol^−1^ for *α* = 0.1, becomes 180 kJ·mol^−1^ for *α* = 0.3 and finally reaches 230 kJ·mol^−1^ for *α* = 0.9 [33]. The decrease in the activation energy is attributed to the influence of CaO on the stiff PC substrate, since this catalyst has no effect on styrenic materials or PP with respect to acceleration of degradation reactions [54].

Blend B/silicalite starts with low values of *E_a_*, at 86 for KAS and 97 kJ·mol^−1^ for the Friedman method for *α* = 0.1; it then increases until reaching a maximum value of 156 and 199 kJ·mol^−1^, respectively, for *α* = 0.4. Afterwards, from *α* = 0.5 to *α* = 0.7, it increases again and shows a second maximum value of 206 for the KAS method and 216 kJ·mol^−1^ for the Friedman isoconversional method. These observations may confirm that the degradation of Blend B/silicalite occurs in two steps, as it was mentioned previously. As regards the catalyst’s effect, it enhances the reduction in *E_a_* required for the degradation. Specifically, it should be mentioned that for the KAS method and without silicalite, *E_a_* was 90 kJ·mol^−1^ at the beginning for *α* = 0.1, and in the end, for *α* = 0.9, it became 230 kJ·mol^−1^ [33], whereas now, in its presence, it is 86 kJ·mol^−1^ when *α* = 0.1 and finally becomes 183 kJ·mol^−1^ when *α* = 0.9.

Once comparing the two blends, the *E_a_* values demanded for Blend B are lower than those of Blend A in every case, plus the degradation starts immediately without the plateau noticed in Blend A (Figure 5). PVC is the polymer that is contained in Blend B and deteriorates its thermal endurance since it is more sensitive with HCl abstraction, resulting in the destabilisation of the blend. Therefore, the abstraction of chlorine atoms from macromolecular chains leads to a much easier initiation of the general degradation of other parts of the polymer in the blend.

All in all, the laboratory-scale experiments of the TGA instrument give valuable details on the temperature and required energies for all types of WEEE polymers studied. We know that fuel-like and aromatic-enriched products in the gasoline range can be produced under catalytic degradation [34], due to chain scission and unzipping as the main degradation mechanisms. The small differences in values accumulated or a detailed evaluation of the results and values may be of little interest to the large-scale application of these catalytic systems, but at the lab-scale, we find them useful for trend mapping. Synergistically, the results show that faster pyrolysis and higher degradation temperatures tend to decrease the molecular weight in the fuel range. Thus, the catalytic pyrolysis of waste polymers and plastics using various catalysts at high heating rates and temperatures appears to be more economically favourable in terms of energy demands.

## 5. Conclusions

In this work, the effect of specific catalysts on the kinetics of the thermal degradation of several polymers and blends was investigated. TGA experiments were carried out for the following combinations of polymers or blends and catalysts: ABS/Fe_2_O_3_, HIPS/Al-MCM-41, PC/Al_2_O_3_, Blend A (=ABS, HIPS, PC and PP)/CaO and Blend B (=ABS, HIPS, PC, PP and PVC)/silicalite. The decomposition kinetics were studied, and the activation energy of each decomposition was calculated using the KAS and Friedman isoconversional methods. Next, comparisons with corresponding data obtained by non-catalytic pyrolysis took place. According to the DTG curves, all polymer/catalyst combinations exhibited one degradation step. Nevertheless, in the case of the blend/catalyst experiments, double-step degradation took place. It was found that the catalysts chosen in all the samples examined were efficient in accelerating the degradation since the maximum rate of degradation appeared at lower temperatures compared to the non-catalytic decompositions. In all cases, the Friedman calculations led to slightly higher *E_a_* values than the KAS method. The presence of the examined catalysts promoted a reduction in the activation energies, verifying in that way their key-role. 

Our results may offer insight for future studies on plastic recycling and WEEE management. The catalysts suggested here may be applied in catalytic pyrolysis of real WEEE in order to reduce energy consumption as an environmentally friendly approach for their management. Having detailed knowledge of the degradation kinetics and mechanisms of such blends will help in the better design of large-scale processes for the recycling of plastics found in WEEE and will provide more gaseous or liquid fractions and less char/residues. The energy demands for these treatments are surely of great importance.

## Figures and Tables

**Table 1:** Information on degradation temperatures and mass loss for the initiation (in), peak (peak) and end (fin) of the catalytic pyrolyses of all substrates used.

Sample	*β* (°C·min^−1^)	T_in_ (°C)	m_in_ (%)	T_peak_ (°C)	m_peak_(%)	T_fin_ (°C)	m_fin_ (%)
ABS/Fe_2_O_3_	20	211	99.23	439	67.85	560	36.09
	10	174	99.54	429	60.33	596	35.40
	5	145	99.78	424	62.92	571	33.96
	2.5	142	99.34	404	66.15	537	30.54
HIPS/Al-MCM-41	20	278	98.01	448	52.60	619	32.46
	10	237	98.03	433	46.71	560	31.69
	5	228	96.31	428	46.08	481	30.38
	2.5	227	97.74	416	42.39	464	29.69
PC/Al_2_O_3_	20	301	97.24	473	62.81	635	37.71
	10	281	98.20	481	53.95	674	32.85
	5	286	97.29	465	59.41	603	37.18
	2.5	274	97.18	402	70.40	582	35.97
Blend A/CaO	20	167	99.96	292, 439	90.32, 60.46	584	36.01
	10	152	99.45	270, 421	89.16, 59.67	539	34.22
	5	116	99.72	262, 404	87.37, 60.10	512	33.03
	2.5	107	99.92	245, 401	86.82, 56.71	492	31.33
Blend B/silicalite	20	163	99.51	309, 440	84.94, 50.24	530	35.24
	10	146	99.55	287, 440	84.27, 52.32	522	35.00
	5	136	99.44	272, 423	84.08, 50.73	511	35.44
	2.5	114	99.93	248, 447	85.38, 55.80	505	34.66

## Data Availability

The original contributions presented in the study are included in the article, further inquiries can be directed to the corresponding author.

## References

[1] Parajuly K., Habib K., Liu G. (2017). Waste electrical and electronic equipment (WEEE) in Denmark: Flows, quantities and management. Resour. Conserv. Recycl..

[2] Charitopoulou M.A., Kalogiannis K.G., Lappas A.A., Achilias D.S. (2021). Novel trends in the thermo-chemical recycling of plastics from WEEE containing brominated flame retardants. Environ. Sci. Pollut. Res..

[3] Achilias D. (2012). Material Recycling-Trends and Perspectives.

[4] Yang X., Sun L., Xiang J., Hu S., Su S. (2013). Pyrolysis and dehalogenation of plastics from waste electrical and electronic equipment (WEEE): A review. Waste Manag..

[5] Nnorom I.C., Osibanjo O. (2008). Sound management of brominated flame retarded (BFR) plastics from electronic wastes: State of the art and options in Nigeria. Resour. Conserv. Recycl..

[6] Siddiqui M.N., Antonakou E.V., Redhwi H.H., Achilias D.S. (2019). Kinetic analysis of thermal and catalytic degradation of polymers found in waste electric and electronic equipment. Thermochim. Acta.

[7] Ma C., Yu J., Wang B., Song Z., Xiang J., Hu S., Su S., Sun L. (2016). Chemical recycling of brominated flame retarded plastics from e-waste for clean fuels production: A review. Renew. Sustain. Energy Rev..

[8] European Commission (2019). https://ec.europa.eu/environment/waste/weee/index_en.htm.

[9] Ma C., Yu J., Wang B., Song Z., Xiang J., Hu S., Su S., Sun L. (2017). Catalytic pyrolysis of flame retarded high impact polystyrene over various solid acid catalysts. Fuel Process. Technol..

[10] Buekens A., Yang J. (2014). Recycling of WEEE plastics: A review. J. Mater. Cycles Waste Manag..

[11] Al-Salem S.M., Antelava A., Constantinou A., Manos G., Dutta A. (2017). A review on thermal and catalytic pyrolysis of plastic solid waste (PSW). J. Environ. Manag..

[12] Rahimi A., García J.M. (2017). Chemical recycling of waste plastics for new materials production. Nat. Rev. Chem..

[13] Sahin O., Kirim Y., Dincer I. (2018). Material recycling. Comprehensive Energy Systems.

[14] Vinu R., Ojha D.K., Nair V., Reedijk J. (2016). Polymer pyrolysis for resource recovery. Reference Module in Chemistry, Molecular Sciences and Chemical Engineering.

[15] Miandad R., Barakat M.A., Aburiazaiza A.S., Rehan M., Nizami A.S. (2016). Catalytic pyrolysis of plastic waste: A review. Process Saf. Environ. Prot..

[16] Jamradloedluk J., Lertsatitthanakorn C. (2014). Characterization and utilization of char derived from fast pyrolysis of plastic wastes. Procedia Eng..

[17] Sharuddin S.D.A., Abnisa F., Daud W.M.A.W., Aroua M.K. (2016). A review on pyrolysis of plastic wastes. Energy Convers. Manag..

[18] Zhao R., Chen J., Liu J., Fan J., Du J. (2015). Morphologies-controlling synthesis of silicalite-1 and its adsorption property. Mater. Lett..

[19] Cesteros Y., Haller G.L. (2001). Several factors affecting Al-MCM-41 synthesis. Microporous Mesoporous Mater..

[20] Naik S.P., Bui V., Ryu T., Miller J.D., Zmierczak W. (2010). Al-MCM-41 as methanol dehydration catalyst. Appl. Catal. A Gen..

[21] Kouzu M., Fujimori A., Suzuki T., Koshi K., Moriyasu H. (2017). Industrial feasibility of powdery CaO catalyst for production of biodiesel. Fuel Process. Technol..

[22] López A., De Marco I., Caballero B.M., Laresgoiti M.F., Adrados A., Aranzabal A. (2011). Catalytic pyrolysis of plastic wastes with two different types of catalysts: ZSM-5 zeolite and Red Mud. Appl. Catal. B Environ..

[23] Samain L., Jaworski A., Edén M., Ladd D.M., Seo D.K., Garcia-Garcia F.J., Häussermann U. (2014). Structural analysis of highly porous *γ*-Al_2_O_3_. J. Solid State Chem..

[24] Kowalska E., Radomska J., Konarski P., Diduszko R., Oszczudłowski J., Opalińska T., Więch M., Duszyc Z. (2006). Thermogravimetric investigation ofwastes from electrical and electronic equipment (WEEE). J. Therm. Anal. Calorim..

[25] De Marco I., Caballero B.M., Chomón M.J., Laresgoiti M.F., Torres A., Fernández G., Arnaiz S. (2008). Pyrolysis of electrical and electronic wastes. J. Anal. Appl. Pyrolysis.

[26] Moltó J., Font R., Gálvez A., Conesa J.A. (2009). Pyrolysis and combustion of electronic wastes. J. Anal. Appl. Pyrolysis.

[27] Siddiqui M.N., Redhwi H.H., Antonakou E.V., Achilias D.S. (2018). Pyrolysis mechanism and thermal degradation kinetics of poly (bisphenol A carbonate)-based polymers originating in waste electric and electronic equipment. J. Anal. Appl. Pyrolysis.

[28] Apaydin-Varol E., Polat S., Pütün A. (2014). Pyrolysis kinetics and thermal decomposition behavior of polycarbonate-a TGA-FTIR study. Therm. Sci..

[29] Janković B. (2009). A kinetic study of the isothermal degradation process of Lexan^®^ using the conventional and Weibull kinetic analysis. J. Polym. Res..

[30] Durmuş A., Koç S.N., Pozan G.S., Kaşgöz A. (2005). Thermal-catalytic degradation kinetics of polypropylene over BEA, ZSM-5 and MOR zeolites. Appl. Catal. B Environ..

[31] Vouvoudi E.C., Achilias D.S., Sideridou I.D. (2015). Dental light-cured nanocomposites based on a dimethacrylate matrix: Thermal degradation and isoconversional kinetic analysis in N_2_ atmosphere. Thermochim. Acta.

[32] Pielichowski K., Njuguna J. (2005). Thermal Degradation of Polymeric Materials.

[33] Roussi A.T., Vouvoudi E.C., Achilias D.S. (2020). Pyrolytic degradation kinetics of HIPS, ABS, PC and their blends with PP and PVC. Thermochim. Acta.

[34] Vouvoudi E.C., Rousi A.T., Achilias D.S. (2023). Effect of the catalyst type on pyrolysis products distribution of polymer blends simulating plastics contained in waste electric and electronic equipment. Sustain. Chem. Process..

[35] Dimitrakakis E., Janz A., Bilitewski B., Gidarakos E. (2009). Small WEEE: Determining recyclables and hazardous substances in plastics. J. Hazard. Mater..

[36] Freegard K., Gayle Tan G., Morton R. (2006). Develop a Process to Separate Brominated Flame Retardants from WEEE Polymers.

[37] Antonakou E.V., Kalogiannis K.G., Stefanidis S.D., Karakoulia S.A., Triantafyllidis K.S., Lappas A.A., Achilias D.S. (2014). Catalytic and thermal pyrolysis of polycarbonate in a fixed-bed reactor: The effect of catalysts on products yields and composition. Polym. Degrad. Stab..

[38] Vyazovkin S., Burnham A.K., Criado J.M., Pérez-Maqueda L.A., Popescu C., Sbirrazzuoli N. (2011). ICTAC Kinetics Committee recommendations for performing kinetic computations on thermal analysis data. Thermochim. Acta.

[39] Vyazovkin S., Sbirrazzuoli N. (2006). Isoconversional kinetic analysis of thermally stimulated processes in polymers. Macromol. Rapid Commun..

[40] Doyle C.D. (1962). Estimating isothermal life from thermogravimetric data. J. Appl. Polym. Sci..

[41] Flynn J.H., Wall L.A. (1966). Thermal analysis of polymer by thermogravimetric analysis. J. Res. Natl. Bur. Stand. A.

[42] Ozawa T. (1965). A new method of analyzing thermogravimetric data. Bull. Chem. Soc. Jpn..

[43] Coats A.W., Redfern J.P. (1964). Kinetic parameters from thermogravimetric data. Nature.

[44] Jang B.N., Wilkie C.A. (2004). A TGA/FTIR and mass spectral study on the thermal degradation of bisphenol A polycarbonate. Polym. Degrad. Stab..

[45] La Mantia F.P., Morreale M., Botta L., Mistretta M.C., Ceraulo M., Scaffaro R. (2017). Degradation of polymer blends: A brief review. Polym. Degrad. Stab..

[46] Madorsky S.L. (1953). Rates and Activation Energies of Thermal Degradation of Styrene and Acrylate Polymers in a Vacuum. J. Polym. Sci..

[47] Liu H., Kong Q., Cheng Y., Cao G. (2012). Thermal Decomposition Kinetics of High Impact Polystyrene/Organo Fe-montmorillonite Nanocomposites. Chin. J. Chem..

[48] Katančić Z., Grčić I., Hrnjak-Murgić Z. (2017). Kinetic Study of Thermal Degradation of High-impact Polystyrene Nanocomposites with Different Flame Retardants using Isoconversional and Model Fitting Methods. Croat. Chem. Acta.

[49] Chrissafis K., Pavlidou E., Vouvoudi E., Bikiaris D. (2014). Decomposition kinetic and mechanism of syndiotactic polystyrene nanocomposites with MWCNTs and nanodiamonds studied by TGA and Py-GC/MS. Thermochim. Acta.

[50] Vyazovkin S. (2015). Isoconversional Kinetics of Thermally Stimulated Processes.

[51] Charde S.J., Sonawane S.S., Sonawane S.H., Navin S. (2018). Influence of functionalized calcium carbonate nanofillers on the properties of melt-extruded polycarbonate composites. Chem. Eng. Commun..

[52] Dong Q., Gao C., Ding Y., Wang F., Wen B., Zhang S., Wang T., Yang M. (2012). A polycarbonate/magnesium oxide nanocomposite with high flame retardancy. J. Appl. Polym. Sci..

[53] Feng Y., Wang B., Wang F., Zhao Y., Liu C., Chen J., Shen C. (2014). Thermal degradation mechanism and kinetics of polycarbonate/silica nanocomposites. Polym. Degrad. Stabil..

[54] Vouvoudi E., Achilias D. (2019). Pyrolytic degradation of common polymers present in packaging materials. J. Therm. Anal. Calorim..

